# Knock-down of ZBED6 in insulin-producing cells promotes N-cadherin junctions between beta-cells and neural crest stem cells *in vitro*

**DOI:** 10.1038/srep19006

**Published:** 2016-01-11

**Authors:** Xuan Wang, Beichen Xie, Yu Qi, Ola Wallerman, Svitlana Vasylovska, Leif Andersson, Elena Nickolaevna Kozlova, Nils Welsh

**Affiliations:** 1 Science for Life Laboratory, Department of Medical Cell Biology, Uppsala University, 751 23 Uppsala, Sweden; 2 Department of Neuroscience, Uppsala University, 751 23 Uppsala, Sweden; 3 Department of Medical Biochemistry and Microbiology, Science for Life Laboratory, Uppsala University, 751 23 Uppsala, Sweden

## Abstract

The role of the novel transcription factor ZBED6 for the adhesion/clustering of insulin-producing mouse MIN6 and βTC6 cells was investigated. *Zbed6*-silencing in the insulin producing cells resulted in increased three-dimensional cell-cell clustering and decreased adhesion to mouse laminin and human laminin 511. This was paralleled by a weaker focal adhesion kinase phosphorylation at laminin binding sites. *Zbed6*-silenced cells expressed less E-cadherin and more N-cadherin at cell-to-cell junctions. A strong ZBED6-binding site close to the N-cadherin gene transcription start site was observed. Three-dimensional clustering in *Zbed6*-silenced cells was prevented by an N-cadherin neutralizing antibody and by N-cadherin knockdown. Co-culture of neural crest stem cells (NCSCs) with *Zbed6*-silenced cells, but not with control cells, stimulated the outgrowth of NCSC processes. The cell-to-cell junctions between NCSCs and βTC6 cells stained more intensely for N-cadherin when *Zbed6*-silenced cells were co-cultured with NCSCs. We conclude that ZBED6 decreases the ratio between N- and E-cadherin. A lower N- to E-cadherin ratio may hamper the formation of three-dimensional beta-cell clusters and cell-to-cell junctions with NCSC, and instead promote efficient attachment to a laminin support and monolayer growth. Thus, by controlling beta-cell adhesion and cell-to-cell junctions, ZBED6 might play an important role in beta-cell differentiation, proliferation and survival.

We have recently observed that the zinc finger BED domain-containing protein 6 (ZBED6) is expressed in insulin-producing cells and that it functions as a transcriptional regulator^1^. This recently discovered transcription factor is unique to and highly conserved among all placental mammals^2^. It belongs to the BED domain-containing family, named after the chromatin-boundary-element-binding proteins BEAF and DREF^3^, and contains two BED domains and a hATC dimerization domain, a feature characteristic of the *hobo*-*Ac*-*Tam3* transposase superfamily^4^. The presence of both BED and hATC domains indicates a transposon-derived origin and the high sequence conservation of the two domains in all placental mammals but not in marsupials suggests that *Zbed6* has evolved an essential function after the split between marsupials and Eutherian mammals^2^,^5^.

The levels of nuclear expression of ZBED6 in insulin producing cells appears to correlate well with proliferation rates in that rapidly proliferating beta-cell lines express high levels of Zbed6 and non-proliferating human islet cells low levels^1^. In addition, we have also observed that down-regulation of ZBED6 in rapidly proliferating cells resulted in lower proliferation rates and increased insulin production, indicating that ZBED6 is inversely correlated to a mature beta-cell phenotype^1^. This prompted us to propose that ZBED6 is expressed during development to maintain proliferation and prevent premature differentiation^1^. ZBED6 is known to act as a transcriptional repressor of the *Igf2* gene^2^, which indicates that it may preferentially bind to and down-regulate genes that mediate cell cycle arrest and efficient insulin production.

Adhesion to extracellular matrix components and cell-to-cell contacts are known to be important for beta-cell embryogenesis, differentiation, proliferation and survival^6^. In our previous study we observed that *Zbed6*-silencing of βTC6 cells resulted in an altered morphology during *in vitro* culture, indicating that ZBED6 affects beta-cell adhesion and cell-to-cell contacts. We have also observed that direct cell-to-cell contacts between beta-cells and neural crest stem cells (NCSCs) promote beta-cell survival^7^ and co-transplantation of islets with NCSCs increases beta-cell proliferation^8^. Therefore, the aim of the present study was to further investigate the role of *Zbed6* in insulin-producing cell adhesion/contact events, using mouse MIN6 and βTC6 cells, and to evaluate the effects of *Zbed6* knockdown on the ability of beta-cells to interact with mouse NCSCs.

## Results

### Stable *Zbed6*-silencing in βTC6 and MIN6 cells

**Figure 1:**
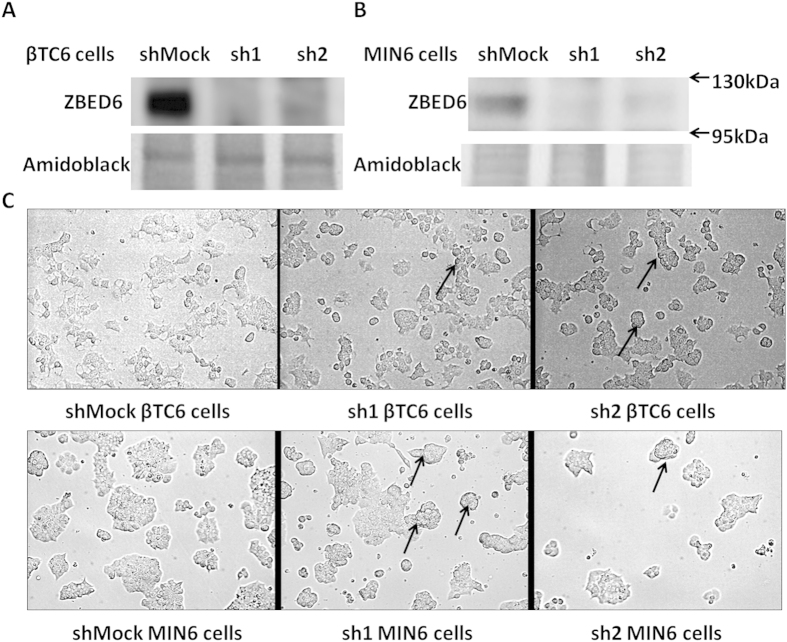
Stable *Zbed6* knockdown-induced morphological changes in βTC6 and MIN6 cells. βTC6 and MIN6 cells were transduced with either *Zbed6* (sh1 and sh2) or mock shRNA lentiviral vectors. ZBED6 protein expression in βTC6 (**A**) and MIN6 (**B**) cells was examined by immunoblot; amidoblack staining for total protein was used as loading control. (**C**) Morphology of βTC6 and MIN6 cells after 3 days of culture; equal numbers of βTC6 or MIN6 cells were seeded to NUNC plastic culture plates without any coating. Arrowheads point to the three-dimensional cell clusters observed in sh1 and sh2 cells, but not in shMock cells. Pictures were taken with a 20X objective.

We achieved stable silencing of *Zbed6* in βTC6 and MIN6 cells by using lentiviral vectors that express *Zbed6-*specific short hairpin RNA (shRNA) (Fig. 1A+B). To decrease the possibility of off-target effects, two different *Zbed6* shRNA sequences (sh1 and sh2) were used. Furthermore, we recently observed that the effects of sh1- and sh2-mediated *Zbed6* knockdown could be reversed by reconstitution of *Zbed6* expression, which strongly indicates that sh1/sh2-induced phenotype occurs via specific *Zbed6* knockdown^1^. A mock lentiviral vector containing a scrambled shRNA sequence was used to generate a negative control cell line (shMock). *Zbed6* silencing was confirmed by Western blotting as efficient suppression of ZBED6 protein expression was observed in both cell lines (Fig. 1A+B).

### *Zbed6*-silenced βTC6 and MIN6 cells display an altered morphology *in vitro*

Plating of an equal number of shMock, sh1 or sh2 cells to culture dishes resulted in an altered organization and morphology of the cells after 3 days of culture. We observed that shMock cells spreaded out and formed traditional monolayers, whereas sh1 or sh2 cells in some cases formed three-dimensional cell clusters (Fig. 1C). These clusters eventually developed into pseudoislet-resembling structures that after many days of culture easily de-attach from the support (results not shown).

### *Zbed6*-silenced βTC6 cells attach less efficiently to a laminin-coated support

**Figure 2:**
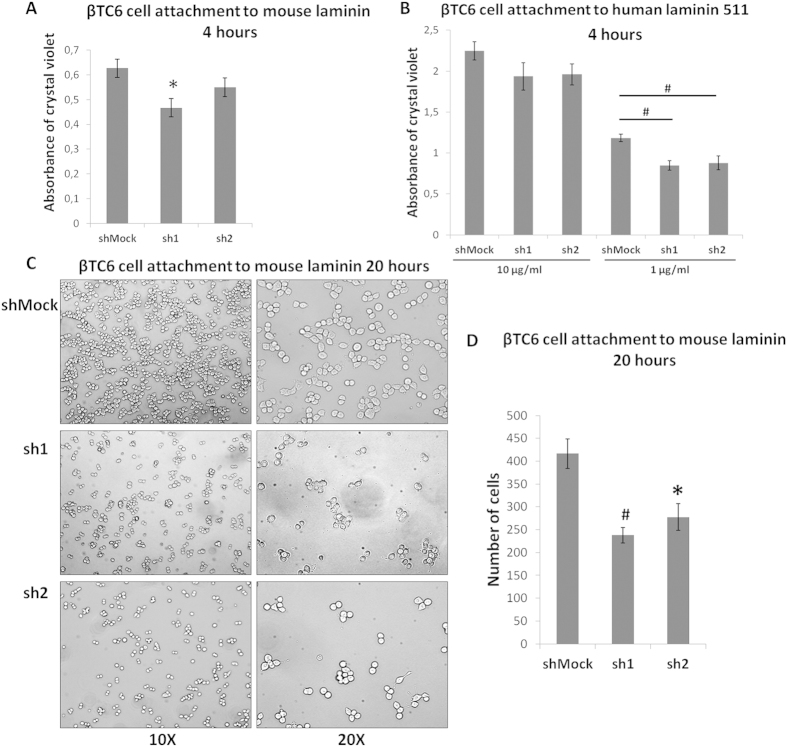
*Zbed6*-knockdown decreases βTC6 cell adhesion to laminin. (**A**) shMock, sh1 and sh2 βTC6 cells were seeded onto mouse laminin (10 μg/ml) coated 96-well plates with the same density (3 × 10^4^ cells per well) and incubated for 4 hours. After removing the non-attached cells, the remaining cells were stained with crystal violet. Results are means ± S.E.M for 4 independent experiments. *denotes P < 0.05 using Student’s t-test. (**B**) shMock, sh1 and sh2 βTC6 cell adhesion to human laminin 511. Study was performed in the same way as (**A**) except that two concentrations of human laminin 511 (10 μg/ml and 1 μg/ml) were used. Results are means ± S.E.M for 4 independent experiments. ^#^denotes P < 0.01 using Student’s t-test. (**C**) Same density of shMock, sh1 and sh2 βTC6 cells were seeded to mouse laminin (10 μg/ml) coated plates and incubated for 20 hours. After removing the non-attached cells, pictures were taken with phase contrast microscope. One representative image of 4 independent experiments is shown, 10X (left panel) and 20X (right panel) objectives were used. (**D**) Analysis of images (10X) from 4 independent experiments by quantifying the number of attached cells using Image J. *denotes P < 0.05, ^#^denotes P < 0.01 using Student’s t-test.

Having observed that *Zbed6*-silenced beta-cells prefer to grow in three-dimensional cell clusters rather than as a monolayer on a plastic support, we next studied whether Zbed6-knockdown affected βTC6 cell attachment to a laminin-coated support. We first investigated attachment to a commercial preparation of mouse laminin, which consists of a mixture of many laminin isoforms, and observed a significantly lower attachment of sh1 cells to this support during a 4 hour incubation period (Fig. 2A). Also sh2 cells tended to bind less efficiently to this support, but this did not reach statistical significance. Next, we studied attachment to human laminin 511, a specific isoform which has been demonstrated to interact with integrin α3β1 of human islet beta-cells and convey proliferation and plasticity of these cells^9^. Also in this case sh1 and sh2 cell adhesion was impaired at 1 μg/ml (Fig. 2B). The adhesion of sh1 and sh2 cells at 10 μg/ml tended to decrease, but the effect was not significant.

We also tested attachment to mouse laminin at 20 hours. Also in this case sh1 and sh2 cell binding to the support was lower as compared to shMock cells (Fig. 2C+D).

### *Zbed6*-silenced βTC6 cells display weaker FAK phosphorylation at sites close to the laminin support

**Figure 3:**
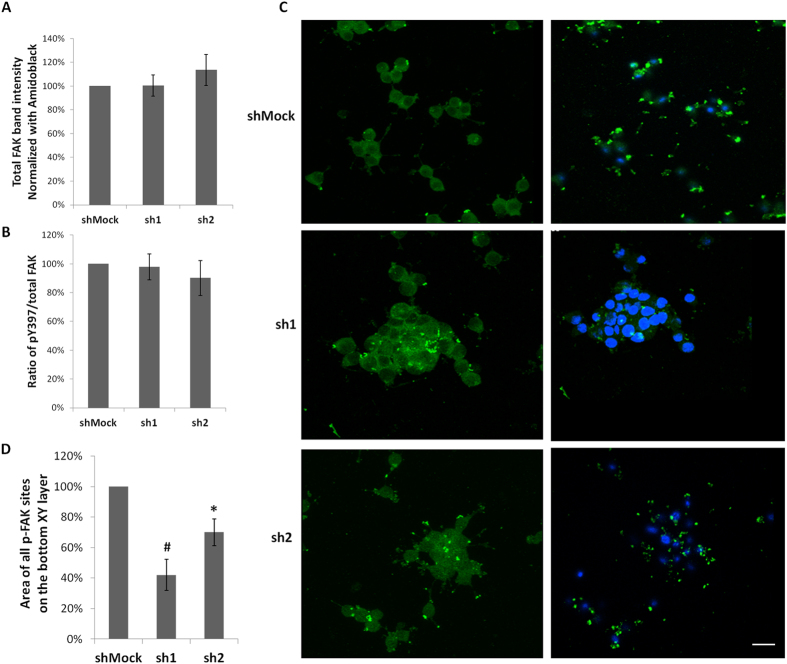
The localization of phospho-FAK was altered by *Zbed6* knockdown in βTC6 cells. (**A**) Equal numbers of shMock, sh1 and sh2 βTC6 cells were seeded onto mouse laminin (10 μg/ml) coated 24-well plates and incubated for 24 hours. The expression of total FAK was determined by immunoblot and normalized to amidoblack staining of total protein. Results are means ± S.E.M for 6 independent experiments. (**B**) The phosphorylation of FAK was examined by immunoblot using the phospho-FAK (Y397) antibody. Ratios of pY397/total FAK were quantified and results are means ± S.E.M for 6 independent experiments. (**C**) Equal numbers of shMock, sh1 and sh2 βTC6 cells were seeded onto mouse laminin (10 μg/ml) coated cover slips and incubated for 24 hours. Cells were stained with a phospho-FAK (Y397) antibody. Images were generated from confocal Z-stack scanning using Imaris Easy 3D model. Left panel: upper XY layer of cells not in direct contact with cover slip. Note the low number of FAK-activation sites in shMock cells, as compared to sh1 or sh2 cells. Right panel: Bottom XY layer of cells close to the cover slip. Note that shMock cells have strong FAK phosphorylation sites whereas sh1 or sh2 cells have weaker and fewer. Results are representative for 3 independent experiments. Scale bar: 20 μm. (**D**) Area of all phospho-FAK sites on the bottom XY layer was quantified by Image J. The results were normalized to the total cell number in each specific image. Results were summarized from 3 independent experiments. *denotes P < 0.05, ^#^denotes P < 0.01 using Student’s t-test.

The weaker adhesion of *Zbed6*-silenced βTC6 cells to laminin supports prompted us to study the Y397 phosphorylation of Focal Adhesion Kinase (FAK), which is a measure of FAK activation that occurs upon cell adhesion to laminin^10^. We observed that neither total FAK levels, nor the total FAK phosphorylation was affected by *Zbed6*-silencing in cells growing on a mouse laminin support (Fig. 3A+B). However, confocal analysis revealed that the distribution of phospho-FAK sites was different in sh1 or sh2 cells as compared to shMock cells. Intercellular phospho-FAK sites were frequent in sh1 or sh2 cells, but not in shMock cells (Fig. 3C). On the other hand, phospho-FAK sites were frequent and prominent close to the laminin support in shMock cells, but not in sh1 or sh2 cells (Fig. 3C+D). Thus, the weaker adhesion to the laminin support of *Zbed6*-silenced cells is associated with a weaker FAK activation at this site.

### *Zbed6*-silenced βTC6 cells form weaker E-cadherin cell-to-cell junctions

**Figure 4:**
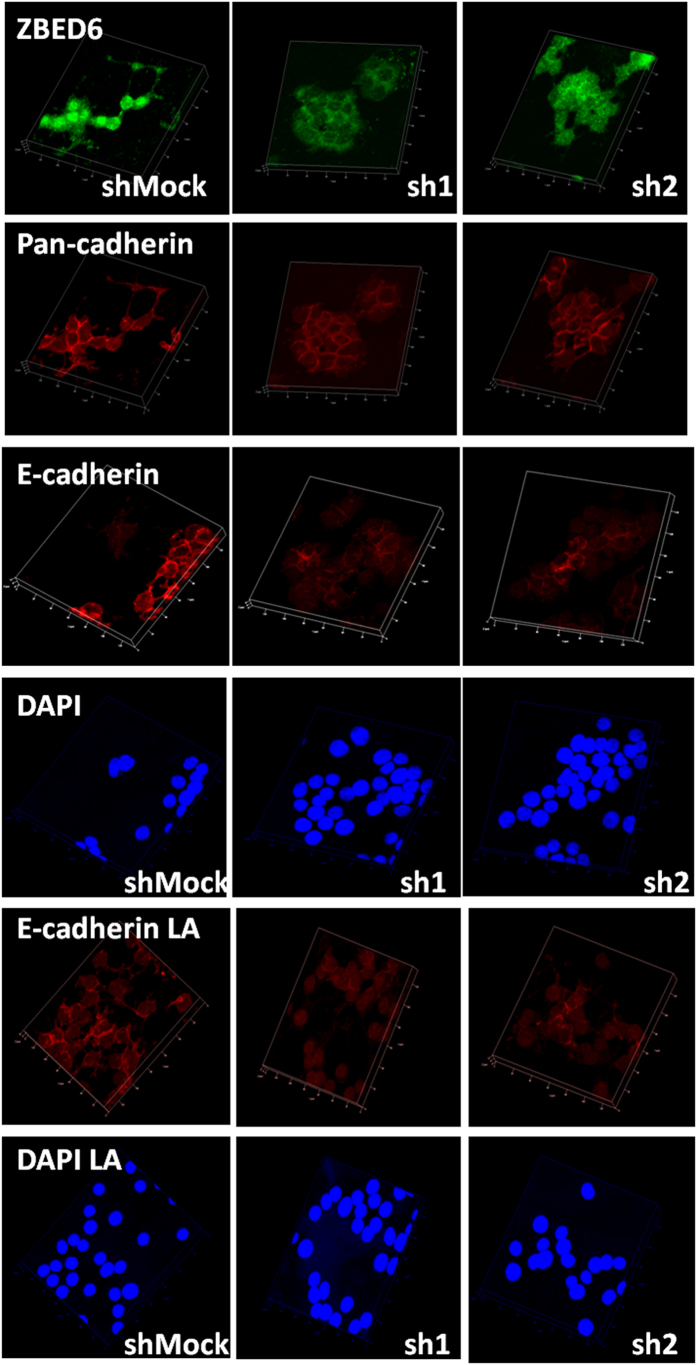
Staining of shMock, sh1 and sh2 βTC6 cells with ZBED6, pan-cadherin and E-cadherin antibodies. Equal numbers of cells were seeded onto cover slips with or without 10 μg/ml mouse laminin (LA) coating. After 3 days culture, cells were fixed and stained. Images were generated from confocal Z-stack scanning using Imaris 3D model. Results are representative for 3 independent experiments. The size of one unit of the XY frame is 20 μm.

We next investigated the effect of *Zbed6* knockdown on beta-cell junctions. Using a pan-cadherin antibody cell-to-cell junctions were visualized three-dimensionally, but no difference in total cadherins between shMock and sh1 or sh2 cells on a plastic support could be observed (Fig. 4). Insulin producing cells are known to express both E-cadherin and N-cadherin^11^. We therefore stained βTC6 cells with an E-cadherin specific antibody. Using this antibody beta-cell junctions were less intensely stained in sh1 or sh2 cells as compared to shMock cells (Fig. 4). Also when grown on a laminin-coated support sh1 or sh2 cells exhibited weaker E-cadherin junctions (Fig. 4).

### *Zbed6*-silenced βTC6 cells form stronger N-cadherin cell-to-cell junctions that are necessary for the three-dimensional growth pattern

**Figure 5:**
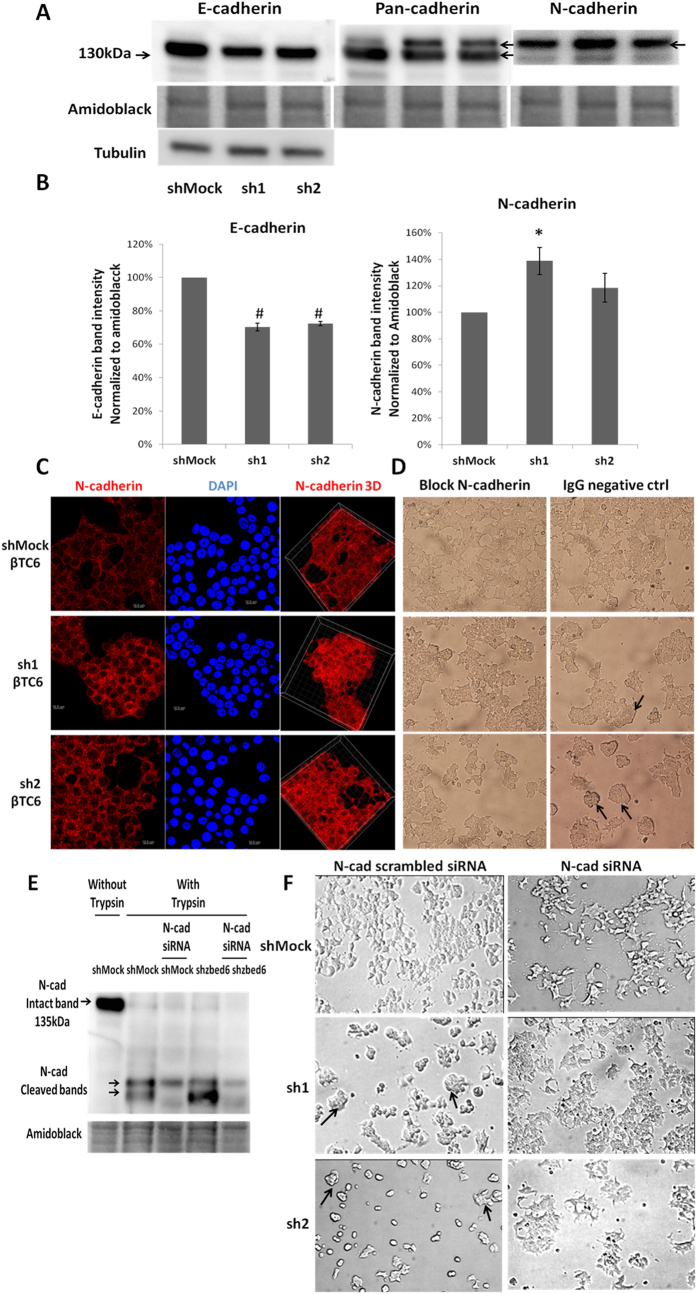
N-cadherin is upregulated in sh1 and sh2 βTC6 cells leading to the formation of three-dimensional cell clusters. (**A**) One representative immunoblot image showing the expression of E-cadherin in shMock, sh1 and sh2 βTC6 cells. Amidoblack was used as a loading control. The molecular weight marker is given on the left. The pan-cadherin antibody recognized two bands on the same blot (upper and lower arrow to the right), of which the lower band is E-cadherin and the upper band N-cadherin. (**B**) Quantification of E and N-cadherin expression in shMock, sh1 and sh2 βTC6 cells. Results are means ± S.E.M for 5 independent experiments. *denotes P < 0.05 and ^#^denotes P < 0.01 using Student’s t-test. (**C**) Staining of N-cadherin in shMock, sh1 and sh2 βTC6 cells. Equal numbers of cells were seeded onto cover slips without laminin coating and incubated for 3 days. Scale bar: 10 μm. (**D**) N-cadherin function was blocked by anti-N-cadherin (mouse IgG1 isotype) antibody. After 2 days incubation, cells were photographed with a 20X objective. Arrowheads point to the three-dimensional cell clusters in the IgG negative control group. Results are representative for 3 independent experiments. PiLenti-siRNA-GFP vectors containing 4 different siRNA sequences all targeting to N-cadherin were transfected to ZBED6-silenced βTC-6 and control cells. GFP positive cells were sorted by FACS. (**E**) The expression of N-cadherin was determined by immunoblot. (Immunoblots were performed directly after trypsinization and sorting therefore the N-cadherin bands were cleaved by trypsin) (**F**) The morphological changes of the sorted GFP positive cells were photographed after 2 days culture without laminin coating (10x objective). Arrowheads point to the three-dimensional cell clusters in the scrambled siRNA group. Results are representative for 3 independent experiments.

Western blot analysis of βTC6 cells confirmed a significant decrease in E-cadherin in sh1 or sh2 cells, as visualized by both the E-cadherin specific antibody and by the pan-cadherin antibody (Fig. 5A+B). N-cadherin, however, which has a slightly higher molecular weight than E-cadherin, was increased in sh1 or sh2 cells (Fig. 5A). This increase reached only statistical significance for sh1 cells (Fig. 5B). The increased expression of N-cadherin was further verified by confocal imaging. Indeed, cell-to-cell N-cadherin junctions were more prominent in the sh1 or sh2 cells than in shMock cells (Fig. 5C). To test the functional importance of the N-cadherin cell-to-cell junctions we plated βTC6 cells in the presence of a N-cadherin neutralizing antibody. Two days after seeding the cells three-dimensional clusters were not observed in the groups cultured in the presence of the N-cadherin antibody, whereas sh1 or sh2 cells that were cultured with control antibody three-dimensional clustering was observed (Fig. 5D). Knockdown N-cadherin by RNAi also hampered the formation of the three-dimensional clusters in the sh1 or sh2 cells after two days culture (Fig. 5E+F).

### *Zbed6*-silenced MIN6 cells form stronger N-cadherin cell-to-cell junction, possibly via a direct transcriptional effect of ZBED6 on the N-cadherin gene

**Figure 6:**
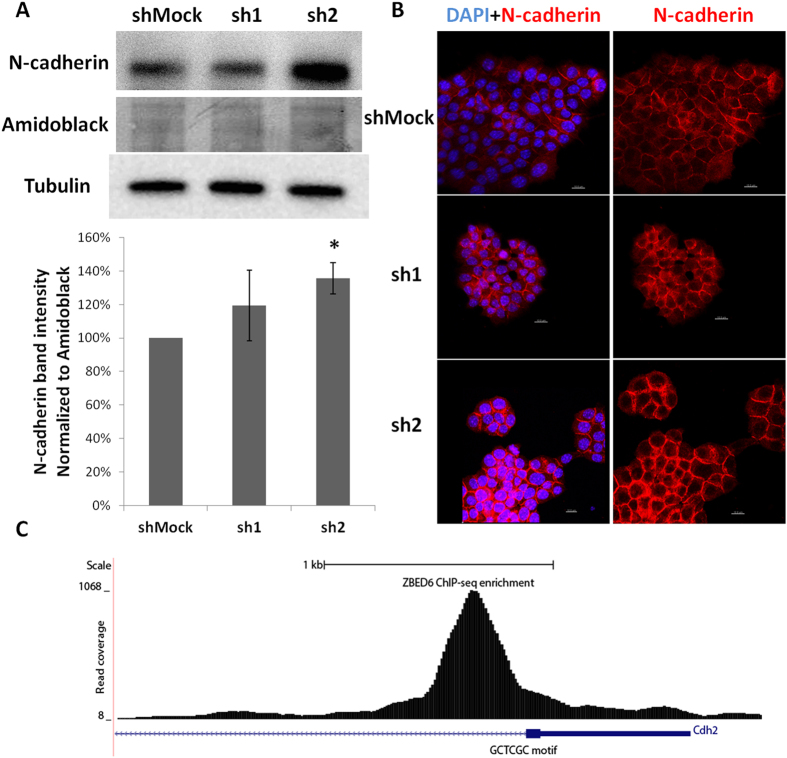
ZBED6 binding to the N-cadherin promoter in MIN6 cells is associated with increased N-cadherin protein levels. (**A**) One representative immunoblot image shows the expression of N-cadherin in shMock, sh1 and sh2 MIN6 cells. Amidoblack was used as a loading control. Quantification of N-cadherin expression in shMock, sh1 and sh2 MIN6 cells. Results are means ± S.E.M for 4 independent experiments. *denotes P < 0.05 using Student’s t-test. (**B**) Staining of N-cadherin in shMock, sh1 and sh2 MIN6 cells. Equal numbers of cells were seeded onto cover slips without laminin coating and incubated for 3 days. Results are representative for 3 independent experiments. Scale bar: 10 μm. (**C**) ChIP sequencing of MIN6 cells using the anti-ZBED6 antibody revealed a strong binding site of ZBED6 close to the Cdh2 (N-cadherin) transcription start site.

Also in MIN6 cells *Zbed6* knockdown resulted in increased N-cadherin protein levels (Fig. 6A). This was paralleled by stronger N-cadherin cell-to-cell junctions as assessed by confocal microscopy analysis (Fig. 6B). To determine whether N-cadherin expression is controlled by ZBED6 via a direct effect on N-cadherin gene transcription we performed ChIP-sequencing using a ZBED6 antibody. Analysis of the N-cadherin gene revealed strong ZBED6 binding approximately 900 bp downstream of the transcription start site (Fig. 6C). This may suggest that ZBED6 directly represses N-cadherin gene transcription.

### The formation of NCSC processes is stimulated by co-culture with sh1 or sh2 βTC6 cells

**Figure 7:**
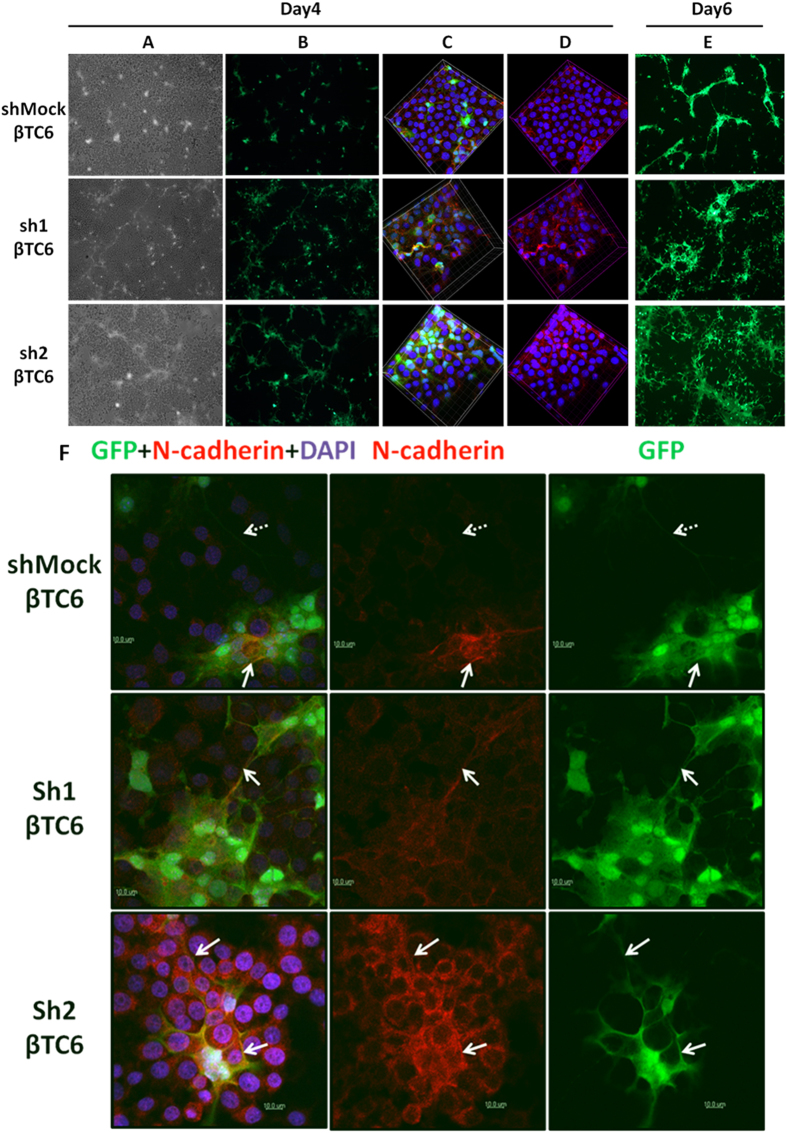
Co-culture of NCSC cells with sh1 and sh2 βTC6 cells results in extensive outgrowth of GFP-positive processes and increased N-cadherin positive NCSC-βTC6 cell junctions. 1 × 10^5^ shMock, sh1 or sh2 βTC6 cells and 2 × 10^5^ GFP-expressing mouse NCSC cells were seeded together onto 10 μg/ml mouse laminin-coated cover slips and kept in culture up to 6 days. (Panel **A**) Phase contrast images show both βTC6 and NCSC cells on day 4 (10X objective). (Panel **B**) NCSC cell GFP signal (green fluorescence) on day 4 showing a weak increase in GFP-positive NCSC processes in sh1 or 2 cells. (Panel **C**) Confocal Z-stack scanning 3D images (63X objective with oil) taken on day 4. Nuclei of both βTC6 and NCSC cells are labeled blue with DAPI. NCSC cells express green GFP fluorescence. Pan-cadherin antibody (red) was used to show the cell-cell junctions and that GFP-positive processes often follow these junctions. (Panel **D**) Images with DAPI and Pan-cadherin staining. (Panel **E**) NCSC cell GFP-signals on day 6 (10X objective) showing extensive outgrowth of NCSC processes during the co-culture with sh1 or 2 cells. Results are representative for 3 independent experiments. (**F**) NCSC-βTC6 cell junctions exhibit increased N-cadherin positivity in sh1 or 2 cells. 1 × 10^5^ shMock, sh1 or sh2 βTC6 cells and 2 × 10^5^ NCSC cells were seeded together onto 10 μg/ml mouse laminin coated cover slips and kept in culture for 4 days. Solid arrows point to the process-to-cell junctions between sh1 or sh2 βTC6 and NCSCs where strong N-cadherin expression can be seen. Dashed arrows point to a GFP-positive process originating from an NCSC without strong N-cadherin expression. Results are representative for 3 independent experiments. Scale bar: 10 μm.

We have previously reported that co-culture of beta-cells with NCSCs resulted in improved beta-cell survival, and that this was possibly mediated via direct cadherin-mediated cell-to-cell contacts^7^. Because *Zbed6*-silenced cells express more N-cadherin than shMock cells, we next studied the effect of *Zbed6* knockdown on interactions between βTC6 cells and NCSCs. Co-culture of GFP-expressing mouse NCSCs with sh1 or sh2 cells for 4 days revealed a slight increase in GFP-positive cell processes (Fig. 7A+B). These processes radiated from NCSC bodies and projected into the surrounding mass of non-GFP positive βTC6 cells, often following the cadherin cell-to-cell junctions (Fig. 7C). After 6 days of co-culture there was a massive increase in NCSC processes using sh1 or sh2 cells, as compared to shMock cells (Fig. 7D).

### Junctions between βTC6-cells and NCSC bodies and processes stain more strongly for N-cadherin when sh1 or sh2 cells were used during *in vitro* co-culture

To further characterize the interaction between βTC6 cells and NCSCs, we stained for N-cadherin after 4 days of co-culture. Again NCSC GFP-positive processes were more frequent in co-culture with sh1 or sh2 cells than when using shMock βTC6 cells (Fig. 7E). In addition, the GFP-positive NCSC processes stained for N-cadherin to a higher extent when surrounded by sh1 or sh2 cells as compared to shMock cells (Fig. 7E). Also the borders between NCSC bodies and βTC6 cells were more intensely stained for N-cadherin when sh1 or sh2 cells were used during co-culture (Fig. 7E).

### Neutralizing N-cadherin reverts the growth of NCSC processes in sh1 or sh2 βTC6 cells

**Figure 8:**
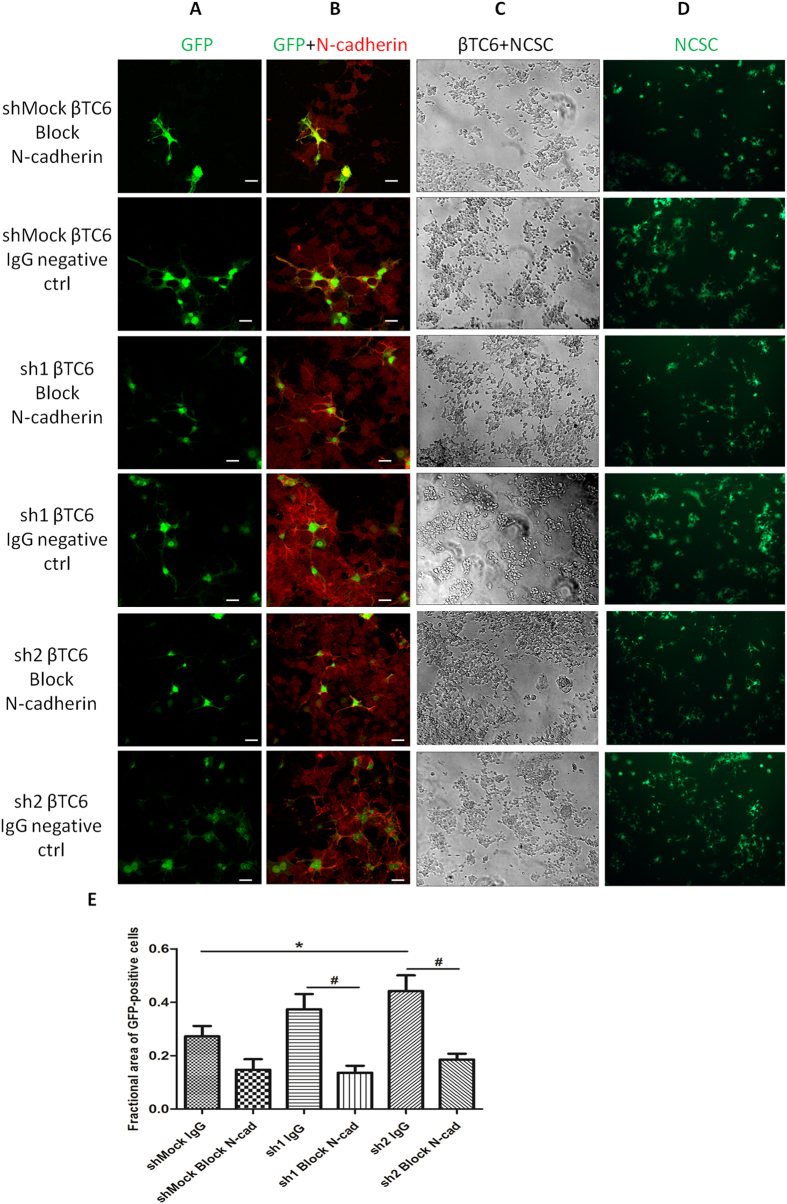
Neutralizing N-cadherin antibody reverts the growth of NCSC processes. 1 × 10^5^ shMock, sh1 or sh2 βTC6 cells and 2 × 10^5^ NCSC cells were seeded together onto 10 μg/ml mouse laminin coated cover slips or culture plates and kept in culture for 4 days supplemented with neutralizing N-cadherin antibody or IgG as a negative control. (Panel **A**) Confocal images (63X objective with oil) of GFP positive NCSC cells. (Panel **B**) Both βTC6 and NCSC cells were stained with anti-N-cadherin antibody (red). NCSC cells express green GFP fluorescence. Scale bar: 10 μm. (Panel **C**) Phase contrast images show both βTC6 and NCSC cells on day 4 (10X objective). (Panel **D**) NCSC cell GFP signal (green fluorescence) on day 4. (**E**) Quantification of fractional area of GFP-positive cells (ratio of GFP-positive cell area to total cell area) by Image J from 4 independent experiments. One-way ANOVA followed by Tukey test was used for multiple comparisons. Statistical significance: *denotes P < 0.05 and ^#^denotes P < 0.01.

To further verify that N-cadherin is necessary for the enhanced growth of NCSC processes when co-cultured with sh1 or sh2 βTC cells, N-cadherin neutralizing antibody was supplemented to the co-culture. After 4 days culture, the N-cadherin positive cell-to-cell junctions between sh1 or sh2 βTC cells as well as the junctions between sh1 or sh2 βTC cells and NCSC cells were stained less intensively in the N-cadherin neutralized group (Fig. 8A+B). After quantifying signals with the Image J software, we observed an increase in the area of NCSC cell bodies and processes co-cultured with sh2 cells and control IgG as compared to shMock cells (Fig. 8C–E). A similar increase was observed with sh1 cells, but in this case it did not reach statistical significance (Fig. 8C–E). The addition of the N-cadherin neutralizing antibody reverted the growth of NCSC processes in sh1 or sh2 βTC cells (Fig. 8C–E).

## Discussion

We presently report that silencing of *Zbed6* resulted in increased protein levels of N-cadherin and decreased levels of E-cadherin. N- and E-cadherin are calcium-dependent glycoproteins that mediate homophilic cell-to-cell contacts, which are important for differentiation, tissue organization, motility, cell polarity, proliferation and survival^12^. We observed also that ZBED6 binds to the N-cadherin promoter region in MIN6 cells, and as it has previously been observed that ZBED6 acts as a repressor of *Igf2* gene transcription^2^, it is possible that ZBED6 also represses N-cadherin gene transcription. Interestingly, we did not observe binding of ZBED6 to the E-cadherin gene (results not shown), suggesting that ZBED6 does not convey a direct effect on beta-cell E-cadherin gene expression. A recent study reported that N-cadherin expression is low and dispensable for beta-cell embryonic development, but that it is necessary for insulin granule turnover and a normal insulin release in adult beta-cells^13^. In addition, N-cadherin has been reported to decrease apoptosis of adult human beta-cells^11^, and to be recruited and activated by secretagogues, thereby promoting a stimulated insulin release^14^. We have recently reported that βTC6 cells, which are rapidly proliferating and have an impaired insulin release as compared to primary adult beta-cells, displayed lower proliferation rates and an increased insulin production in response to *Zbed6* knockdown^1^, and it is possible that the improved phenotype of the βTC6 sh1 or sh2 cells is, in part, mediated by an increased N-cadherin expression. This notion is supported by the present observation that neutralization of N-cadherin cell-to-cell contacts, using a N-cadherin antibody, or by knocking down N-cadherin using an RNAi approach, counteracted the formation of three-dimensional structures often observed during culture of sh1 or sh2 cells. Three-dimensional growth and the formation of pseudo-islet structures is known to enhance beta-cell function as compared to dispersed or mono-layer cells^15^.

In the present investigation *Zbed6* knockdown resulted in the formation of more prominent N-cadherin-containing junctions between beta-cells and NCSCs. We have previously observed that direct contacts between beta-cells and NCSCs increase beta-cell resistance against cytokine-induced cell death^7^. E- and N-cadherin junctions between beta-cells promote improved survival and function^11^, and it is likely that cadherin junctions also between NCSC and βTC6 cells exert similar effects. It is therefore possible that *Zbed6* knockdown-mediated up-regulation of N-cadherin will further enhance beta-cell survival when in contact with surrounding NCSCs, especially as NCSC expression of E-cadherin was not detectable in our experimental setting (results not shown). Interestingly, co-culture of NCSCs with sh1 or sh2 βTC6 cells stimulated NCSC process formation. We have recently observed that these extensions are outgrowths from both neurons, i.e. neurites (axons or dendrites), and glia cells, i.e. glia cell processes (Fred, R. *et al.* manuscript submitted for publication). In both cases, the formation of these processes suggests that the NCSCs differentiate from a stem cell state to a more differentiated state upon co-culture with beta-cells. It has been observed that neural crest cells play an important role in the embryogenesis of the pancreas and its beta-cells, possibly via direct cell-to-cell contacts^16^. More specifically, removal of neural crest cells by *Foxd3* knock-out resulted in increased endocrine proliferation, but also a loss of beta-cells function as indicated by lower insulin, PDX-1 and MafA levels^16^. Thus, direct contact with neural crest cell processes, via N-cadherin cell-to-cell junctions, might enhance beta-cell function/maturation, and beta-cells with higher N-cadherin expression are probably more prone to stimulate the formation of and form contacts with neural crest cell processes.

The reason for the *Zbed6* knockdown-induced reduction of E-cadherin is not known. It could be speculated that increased N-cadherin expression promotes feedback inhibition on the expression of E-cadherin, but this requires further experimental verification. It is also unclear by which mechanisms an altered ratio between N-cadherin and E-cadherin affects beta-cell morphology and interaction with NCSCs. Cadherins are known to recruit and induce intracellular signalling events via different catenins, p120 and GTPases of the Rho family^12^, but the exact differences between N- and E-cadherin-induced signalling in beta-cells have not been delineated. However, our present findings support the notion that an altered N-cadherin to E-cadherin ratio, in βTC6 and MIN6 cells, alters intracellular signalling events so that the interaction with NCSCs and the insulin production is improved. Furthermore, the altered cadherin signalling in sh1 or sh2 cells may also have modified beta-cell adhesion to laminin leading to a lower FAK phosphorylation at contact points to the matrix. A lower FAK activity is compatible with an impaired beta-cell function, but as the sh1 or sh2 cells compensated with more intercellular FAK activation sites, there was no over-all change in FAK activation.

In summary, we report that ZBED6 inhibits N-cadherin gene expression and that this leads to impaired beta-cell three-dimensional growth and interaction with neural crest cells. Strategies aiming at decreasing ZBED6 expression in immature or precursor cells might improve maturation of functional beta-cells in beta-cell replacement trials.

## Materials and Methods

### Cell culture

Murine βTC6 cells, purchased from ATCC, at passage numbers 20–40 were maintained in Dulbecco’s Modified Eagle’s Medium (DMEM) (Gibco) supplemented with 10% fetal calf serum (FCS) (Sigma Chemicals), 2 mM L-glutamine, streptomycin (0.1 mg/ml) and benzylpenicillin (100 U/ml). Murine MIN6 cells (passage 20–35) were maintained in DMEM with 15% fetal calf serum, 2 mM L-glutamine, 70 μM 2-mercaptoethanol, streptomycin (0.1 mg/ml) and benzylpenicillin (100 U/ml). All cells were kept at 37 °C in a humidified atmosphere with 5% CO_2_.

### Generation of stable ZBED6-shRNA βTC6 and MIN6 cell lines

Short-hairpin sequence against the Zbed6 gene were cloned into lentiviral vectors and used for βTC6 and MIN6 cell transduction as previously described^1^. The target sequences selected are: ZBED6-sh1: 5′- CTTCAACACTTCAACGACA -3′; ZBED6-sh2: 5′- TGTGGTACATGCAATCAAA -3′. βTC6 and MIN6 cells were transduced with the shRNA lentiviral particles (10 MOI) and cells with stable expression of shZBED6 were selected by incubation in a medium containing puromycin (10 μg/ml) for at least 2 weeks. Control cells (shMock) were transduced with virus carrying a scrambled shRNA sequence. Multiple cell clones from the shMock, sh1 and sh2 treatments were pooled to generate the three mixed cell populations, respectively, which minimizes the risk of random clonal selection. ZBED6 protein expression was confirmed by immunoblotting.

### Plate coating and cell attachment assay

The culture plates or cover slips were coated with 10 μg/ml mouse laminin (Invitrogen) or 10 μg/ml and 1 μg/ml human laminin 511 (BioLamina) in PBS over night at 4 °C. After laminin coating, the remaining uncoated surfaces were blocked with BSA for 1 hour at 37 °C. The culture plates and cover slips were washes 3 times with PBS before use.

Cells were harvested by Accutase (Millipore). Serum-containing DMEM was used to inactivate Accutase. Cells were then washed with serum free DMEM, counted and allowed to recover in suspension for half an hour at 37 °C. For cell attachment assay, cells (3 × 10^4^ cells per well) were seeded onto the laminin coated 96-well plates and incubated for 4 hours. After removing the non-attached cells, the attached cells were fixed with 96% ethanol for 10 minutes and stained with crystal violet (0.1% in water) for 20 minutes. After extensive washing, the stained protein was solubilized with 0.5% SDS and the absorbance was measured at 600 nm.

### Immunoblot analysis

Cells were washed in cold PBS and lysed on ice in SDS-sample buffer after either 24 hours or 3 days culture. Immunoblot analysis was performed as previously described^1^ using the following antibodies: anti-mouse ZBED6 (1:1000), FAK and phospho-FAK Y397(Cell Signaling), N-cadherin (Abcam 12221) and (MNCD2, DSHB), E-cadherin (Abcam 76055), pan-cadherin (Abcam 6528), alpha-tubulin (Santa Cruz 8035). Total protein loading and transfer onto the membranes was visualized by amidoblack staining. In some experiment, alpha-tubulin was also used as a loading control.

### Immunofluorescence and confocal microscopy

βTC6 or MIN6 cells were cultured on uncoated or laminin coated coverslips for 1–4 days before staining. Cells were fixed in 4% PFA for 10 min at room temperature, permeabilized with 0.2% Triton X-100 on ice for 10 min, blocked with 5% FCS for 30 min in PBS and then incubated for 1 h with antibodies at room temperature. The cells were then washed four times with PBS to remove unbound antibodies and then treated with Alexa Fluor 488-labeled goat anti-rabbit, 594-labeled goat anti-mouse, and 568-labeled goat anti-rabbit secondary antibodies (20 μg/ml each) (Life Technologies) for 1 h. Cells were washed four times with PBS and mounted with VECTASHIELD Hard Set mounting medium with DAPI (Vector Laboratories) and inspected with a Zeiss 780 confocal microscope.

### Inhibition of N-cadherin-mediated cell adhesion

The monoclonal anti-N-cadherin (clone GC-4, Sigma) antibody (50 μg/ml) was used to functional inhibit N-cadherin-mediated cell adhesion. Non-immune IgG (50 μg/ml) was used as a negative control. PiLenti-siRNA-GFP vectors (abm) containing 4 different siRNA sequences all targeting to N-cadherin (AAGGATGTGCACGAAGGACAG; AAGCCACAGACATGGAAGGCA; ACTGTGTCTGTGACAGTTATTGATGTCAA; TTGTCAGTGTGACTCCAATGGAGACTGC) were also used to knockdown N-cadherin in ZBED6-silenced βTC-6 and control cells. After transfection, the GFP positive cells were sorted using the Becton Dickinson FACSCalibur flow cytometer by gating the cell population with increased FL-1 channel signal intensity (GFP-fluorescence). Sorted cells (10^5^) were concentrated by centrifugation and then analyzed by Western blot.

### ChIP-seq analysis

MIN6 cells were crosslinked with 1% formaldehyde for 10 min, quenched with glycine and stored at −80 °C. After thawing and treatment with cell lysis buffer, chromatin was sonicated in RIPA buffer using a BioRuptor (40 min with 30 s on/off cycles in 2 × 750 μl buffer). Two separate ChIPs were prepared using chromatin from 66 million and 10 million cells respectively using 20 μl Dynal protein G beads with 2 μg ZBED6 antibody. Illumina libraries were prepared using NEXTflex adaptors (BIOO Scientific) and enzymes from Fermentas (Fast End Repair, 25 μl for 15 min, 1 μl Klenow exo-minus DNA polymerase for 30 min at 37 °C, 0.5 μl fast ligase for 15 min). Sequencing was done using Illumina HiSeq 2000 instruments. Genomic reads from MIN6 cells were downloaded from Array Express (E-MTAB-1143) and used as a control in peak calling. All reads were aligned to the mouse mm9 assembly using BWA version 0.5.9 at default settings. SAMtools^17^ were used to remove alignments with low alignment quality (<20) and the MACS peak caller (version 1.41) was used to identify enriched peaks and create wiggle tracks for visualization. Since both replicates showed strong enrichment and the duplication rate was low outside of peaks, the final peak calling was done on the combined dataset by keeping all duplicates to avoid saturated peaks. Motif analysis was done with MEME-ChIP^18^ on the 200 bp sequences centered on the peak summits for the 500 highest and 500 lowest peaks. Default settings were used except that the search space was limited to motifs of 6–20 bp length.

### Mice

Transgenic heterozygous C57BL/6-β-actin enhanced green fluorescent protein (EGFP) mice (Jackson Laboratories, Bar Harbor, ME, http://www.jax.org) were used to generate NCSCs. All procedures were approved by the Regional Ethics Committee for Research on Animals (The Uppsala County Regional Ethics Committee for Research on Animals) and carried out in accordance with the approved guidelines.

### Preparation of NCSC and βTC6 cells co-culture

Dorsal root ganglia from E11.5 day old EGFP mouse embryos were isolated and used to generate NCSC neurospheres (NL38 cell line) from the so-called boundary cap^19^,^20^. The neurospheres were treated with collagenase/dispase and plated in 24-well plates as previously described^7^. After 12 hours non-adherent cells were collected and further cultured for formation of neurospheres. The cells were maintained in DMEM medium with supplement of N2, B27, FGF (20ng/ml) and EGF (20ng/ml). The neurospheres were then collagenase treated for co-culture of single NCSCs with βTC6 cells. For the co-culture, 1 × 10^5^ shMock, sh1 or sh2 βTC6 cells and 2 × 10^5^ NCSC cells were seeded together onto 10 μg/ml mouse laminin-coated cover slips and incubated up to 6 days.

### Image analysis and quantification

Image J (http://imagej.net/Home) was used for image quantification. For automatic cell counting, the following method was used: https://digital.bsd.uchicago.edu/docs/cell_counting_automated_and_manual.pdf.For area measurement, the following method was used: https://www.med.upenn.edu/cellbio/documents/ImageJ_ColorSegmentpdf.pdf. Imaris (http://www.bitplane.com/imaris) was used to generate 3D image.

### Statistical analysis

Data are presented as means ± S.E.M. Statistical significance for pairwise comparisons was analyzed using Student’s t-test. One-way ANOVA followed by Tukey test was used for multiple comparisons. Statistical significance: *denotes P < 0.05 and ^#^denotes P < 0.01.

## Additional Information

**How to cite this article**: Wang, X. *et al.* Knock-down of ZBED6 in insulin-producing cells promotes N-cadherin junctions between beta-cells and neural crest stem cells *in vitro*. *Sci. Rep.* **6**, 19006; doi: 10.1038/srep19006 (2016).

## References

[b1] Wang X. *et al.* Transcription factor ZBED6 affects gene expression, proliferation, and cell death in pancreatic beta cells. Proc Natl Acad Sci USA 110, 15997–6002 (2013). 24043816 10.1073/pnas.1303625110 PMC3791784

[b2] Markljung E. *et al.* ZBED6, a novel transcription factor derived from a domesticated DNA transposon regulates IGF2 expression and muscle growth. PLoS Biol 7, e1000256 (2009). 20016685 10.1371/journal.pbio.1000256 PMC2780926

[b3] Aravind L. The BED finger, a novel DNA-binding domain in chromatin-boundary-element-binding proteins and transposases. Trends Biochem Sci 25, 421–3 (2000). 10973053 10.1016/s0968-0004(00)01620-0

[b4] Calvi B. R., Hong T. J., Findley S. D. & Gelbart W. M. Evidence for a common evolutionary origin of inverted repeat transposons in Drosophila and plants: hobo, Activator, and Tam3. Cell 66, 465–71 (1991). 1651170 10.1016/0092-8674(81)90010-6

[b5] Andersson L. *et al.* ZBED6: The birth of a new transcription factor in the common ancestor of placental mammals. Transcription 1, 144–148 (2010). 21326889 10.4161/trns.1.3.13343 PMC3023575

[b6] Otonkoski T., Banerjee M., Korsgren O., Thornell L. E. & Virtanen I. Unique basement membrane structure of human pancreatic islets: implications for beta-cell growth and differentiation. Diabetes Obes Metab 10 Suppl 4, 119–27 (2008). 10.1111/j.1463-1326.2008.00955.x 18834439

[b7] Ngamjariyawat A., Turpaev K., Vasylovska S., Kozlova E. N. & Welsh N. Co-culture of neural crest stem cells (NCSC) and insulin producing beta-TC6 cells results in cadherin junctions and protection against cytokine-induced beta-cell death. PLoS One 8, e61828 (2013). 10.1371/journal.pone.0061828 PMC3629122 23613946

[b8] Grapensparr L. *et al.* Co-transplantation of human pancreatic islets with post-migratory neural crest stem cells increases beta-cell proliferation and vascular and neural regrowth. J Clin Endocrinol Metab 100, E583–90 (2015). 25668197 10.1210/jc.2014-4070

[b9] Banerjee M., Virtanen I., Palgi J., Korsgren O. & Otonkoski T. Proliferation and plasticity of human beta cells on physiologically occurring laminin isoforms. Mol Cell Endocrinol 355, 78–86 (2012). 22314207 10.1016/j.mce.2012.01.020

[b10] Salasznyk R. M., Klees R. F., Boskey A. & Plopper G. E. Activation of FAK is necessary for the osteogenic differentiation of human mesenchymal stem cells on laminin-5. J Cell Biochem 100, 499–514 (2007). 16927379 10.1002/jcb.21074

[b11] Parnaud G. *et al.* Cadherin engagement protects human beta-cells from apoptosis. Endocrinology 152, 4601–9 (2011). 21990317 10.1210/en.2011-1286

[b12] Meda P. Protein-mediated interactions of pancreatic islet cells. Scientifica (Cairo) 2013, 621249 (2013). 24278783 10.1155/2013/621249 PMC3820362

[b13] Johansson J. K. *et al.* N-cadherin is dispensable for pancreas development but required for beta-cell granule turnover. Genesis 48, 374–81 (2010). 20533404 10.1002/dvg.20628 PMC2921608

[b14] Parnaud G. *et al.* Cadherin engagement improves insulin secretion of single human beta-cells. Diabetes 64, 887–96 (2015). 25277393 10.2337/db14-0257

[b15] Hauge-Evans A. C., Squires P. E., Persaud S. J. & Jones P. M. Pancreatic beta-cell-to-beta-cell interactions are required for integrated responses to nutrient stimuli: enhanced Ca^2+^ and insulin secretory responses of MIN6 pseudoislets. Diabetes 48, 1402–8 (1999). 10389845 10.2337/diabetes.48.7.1402

[b16] Plank J. L. *et al.* Influence and timing of arrival of murine neural crest on pancreatic beta cell development and maturation. Dev Biol 349, 321–30 (2011). 10.1016/j.ydbio.2010.11.013 PMC3019241 21081123

[b17] Li H. *et al.* The Sequence Alignment/Map format and SAMtools. Bioinformatics 25, 2078–9 (2009). 10.1093/bioinformatics/btp352 PMC2723002 19505943

[b18] Bailey T. L. *et al.* MEME SUITE: tools for motif discovery and searching. Nucleic Acids Res 37, W202–8 (2009). 19458158 10.1093/nar/gkp335 PMC2703892

[b19] Hjerling-Leffler J. *et al.* The boundary cap: a source of neural crest stem cells that generate multiple sensory neuron subtypes. Development 132, 2623–32 (2005). 15872002 10.1242/dev.01852

[b20] Aldskogius H. *et al.* Regulation of boundary cap neural crest stem cell differentiation after transplantation. Stem Cells 27, 1592–603 (2009). 19544468 10.1002/stem.77 PMC2733376

